# Communication between family carers and health professionals about end-of-life care for older people in the acute hospital setting: a qualitative study

**DOI:** 10.1186/s12904-015-0032-0

**Published:** 2015-08-01

**Authors:** Glenys Caswell, Kristian Pollock, Rowan Harwood, Davina Porock

**Affiliations:** School of Health Sciences, University of Nottingham, Queen’s Medical Centre, Nottingham, UK; Nottingham University Hospitals NHS Trust, Queen’s Medical Centre, Nottingham, UK; School of Nursing, University at Buffalo, 101 C Wende Hall, Buffalo, New York USA

**Keywords:** Acute hospital, Compliance, Concordance, End of life care, Family caregivers, Family carers, Older people, Staff-carer communication

## Abstract

**Background:**

This paper focuses on communication between hospital staff and family carers of patients dying on acute hospital wards, with an emphasis on the family carers’ perspective. The age at which people in the UK die is increasing and many continue to die in the acute hospital setting. Concerns have been expressed about poor quality end of life care in hospitals, in particular regarding communication between staff and relatives. This research aimed to understand the factors and processes which affect the quality of care provided to frail older people who are dying in hospital and their family carers.

**Methods:**

The study used mixed qualitative methods, involving non-participant observation, semi-structured interviews and a review of case notes. Four acute wards in an English University teaching hospital formed the setting: an admissions unit, two health care of older people wards and a specialist medical and mental health unit for older people. Thirty-two members of staff took part in interviews, five members of the palliative care team participated in a focus group and 13 bereaved family carers were interviewed. In all, 245 hours of observation were carried out including all days of the week and all hours of the day. Forty-two individual patient cases were constructed where the patient had died on the wards during the course of the study. Thirty three cases included direct observations of patient care. Interviews were completed with 12 bereaved family carers of ten patient cases.

**Results:**

Carers’ experience of the end of life care of their relative was enhanced when mutual understanding was achieved with healthcare professionals. However, some carers reported communication to be ineffective. They felt unsure about what was happening with their relative and were distressed by the experience of their relative’s end of life care.

**Conclusions:**

Establishing a concordant relationship, based on negotiated understanding of shared perspectives, can help to improve communication between healthcare professionals and family carers of their patients.

## Background

The age at which people die is increasing, with 36.2 % of deaths in England in 2011 occurring in people over 85 years of age [1]. Current English policy promotes death at home as the favoured option [2]. It has been estimated that by 2030 44 % of deaths will be of individuals over the age of 85, and that the number of deaths which occur in acute hospitals will increase from the current 58 % to 65 % [3]. General hospitals are designed to treat and cure acute illness rather than care for dying patients, many of whom are infirm, frail and may be confused. This raises concerns that the hospital environment is inappropriate for the provision of end of life care for patients and their families, and that the quality of end of life care in acute hospitals is sometimes inadequate [4–9]. Communication is a specific aspect of care which is reported as poor [4, 7,10]. A recent review reported that professionals still used incomprehensible language, lacked the skills to deliver bad news and patients and families viewed professionals as being too busy to be available to talk [11]. Poor communication in healthcare settings is not a new issue, as it has long been recognised that discussing death and dying with patients and carers is difficult for healthcare professionals [12]. At the same time it is also recognised that good communication is a vital ingredient of end of life care, and that training in communication skills is necessary [13, 14].

Poor communication between hospital staff and family carers (henceforth carers) can cause distress and dissatisfaction, and is a common topic of NHS complaints [15]. A recent audit of care of the dying in English hospitals carried out by the Royal College of Physicians supports the view that there is room for improvement. For example, in only 57 % of cases was there documented evidence that the plan for care whilst the patient was dying was communicated with the patient and their nominated relative or friend [10]. Lack of effective communication between professionals and patients and carers was also a key finding of the *More Care, Less Pathway* report [16]. The review which preceded this report was prompted by concerns about the implementation of the Liverpool Care Pathway for the Dying Patient (LCP) [17].

At the time of data collection the study hospital used the LCP, an integrated care pathway developed to transfer best practice in the care of dying patients from the hospice to other settings. The LCP was developed during the 1990s, and offered a pathway which could be used to plan individualised care for dying patients. One of its key emphases is on the importance of effective communication and the need to prioritise communication with families when a dying patient is placed on the pathway [18]. The *More Care, Less Pathway* report recommended the withdrawal of the LCP on a number of grounds. One of these was the lack of communication with staff reported by carers as their relatives were dying, as well as a lack of consideration shown by healthcare professionals to both patients and carers towards the end of life [16].

Effective communication is a key component of good end of life care, enabling patients and their family carers to understand what is happening and to adjust to their new situations. Professional bodies regulating medical and nursing practice emphasise the importance of communicating well with patients and carers and of doing so in non-technical language that may be easily understood [19, 20]. However, hospitals are reported to lack formal procedures or requirements for involving carers in decision making. For example, relatives in one United States study complained that they were neither informed nor consulted about treatment decisions for their relative [21].

Delivering bad news to patients and their families is a difficult task especially in the acute hospital setting which presents a challenging environment with limited access to privacy and little time for health professionals to establish a relationship with patients and families [22–24]. When a frail older person is approaching the end of life the physician/patient dyad expands to include carers, who may act as proxy for the patient in cases where the patient is too ill to discuss end of life care options. In addition to knowing *how* to talk to patients and families about approaching death, professionals also need to be able to assess *whether* a particular individual wishes to discuss end of life issues and *when* the time is appropriate to enter into such a discussion [25, 26].

Informing patients and their families that death is expected is often done by a senior clinician, but in practice less senior doctors may take on this responsibility. Nurses usually take the supporting role in this context, for example providing further explanations or clarifications for patients and their carers. However, nurses have also been identified as having a key role in breaking bad news when there is a sudden or unexpected event, such as death being recognised as imminent [27] or death occurring during the night when the family were not present. Communication is, therefore, a key task for all healthcare professionals involved in the care of dying patients.

Communication involves the sending and receiving of messages between two or more individuals during which each participant endeavours to make sense of the interaction and decides how to react and respond [28]. Participants in the process are not equal, however, and in a hospital setting healthcare professionals have more power than patients or their carers. This power is derived from the status and authority that doctors in particular have, based on their professional expertise and the role that they fulfil in the hospital setting [29]. Despite significant attempts through professional education, carers may still find it difficult to raise their worries with clinicians within the context of a conversation which is led by the professional and in which carers are often perceived as the passive recipients of information, rather than as active participants to a shared discussion [28]. This is particularly true in the context of discussing dying.

There has been little research focusing specifically on communication between hospital staff and the family carers of patients [7, 8,30–34]. This paper makes a significant contribution to knowledge of the topic through the discussion of research findings with reference to processes of communication between members of staff and family carers of patients who died on the wards.

## Methods

The study design was qualitative, using an ethnographic approach, utilising mixed methods of non-participant observation, semi-structured interviews with health professionals and bereaved carers, and a review of medical and nursing notes [35].

### Data collection

The setting for the study was four wards in an English university teaching hospital. One ward was an acute admissions ward (Ash; both sexes in separate bays), one was a specialist medical and mental health unit for older people with cognitive impairment (Oak; both sexes in separate bays ) and two were health care of older people wards (one male: Elm, one female: Fir). Across the four wards 245 hours of non-participant observation were carried out on all days of the week and across the 24 hours of the day. Particular attention was paid to interactions between staff and carers, providing a rich source of data to complement interviews with carers on the issue of carer/staff communications. Thirty-two semi-structured interviews were conducted with members of staff, and one focus group was held with five members of the palliative care team. Table 1 shows the characteristics of the wards.

**Table 1:** Ward characteristics

Pseudonym	Oak	Ash	Elm	Fir
Type of ward	Medical and Mental Health Unit (MMHU)	Acute Admissions Unit (AMU)	Health Care of Older People (HCOP) Male patients	Health Care of Older People (HCOP) Female patients
Number of beds	28	42	23	28
Approx. number of staff	45	75	31	38
Number of deceased patients whose notes accessed	17	10	6	9

Ward characteristics

Patient cases were compiled featuring 42 patients who died on the study wards during the course of the research. The care of 33 case patients as they were dying was the focus of observations, and the case notes of these plus a further nine patients who died on the study wards were reviewed, making a total of 42 patients. Their characteristics are shown in Table 2. The family carers of the 39 patient cases who had relatives were invited to take part in an interview, resulting in interviews with 13 bereaved family carers, discussing the end of life care of 11 patients. Inclusion criteria for these interviews included relatives and carers of older patients, so that participants were family members who may, or may not, have provided care for the patient. Each patient case was formed of at least two different data-types including case notes, observations and carer interviews. Approximately ten percent of cases did not have family members who could be invited to participate in an interview. Interviews were conducted by one of two members of the research team.

**Table 2:** Patient characteristics

Ward	Dementia diagnosis	Number of patient cases	Average age of patients (range)	Male patients	Female patients	Average length of stay in days (range)	Patients with DNACPR	Patients on LCP
Oak MMHU	Dementia	11	83	7	4	21 (5–38)	11	9
	No dementia	6	81	4	2	19 (6–50)	5	4
Ash (AMU)	Dementia	2	86	2	0	3 (2–4)	2	1
	No dementia	8	83	4	4	1.5 (1–2)	8	5^a^
Elm HCOP Male	Dementia	2	85	2		10 (5–15)	2	2
	No dementia	4	83.5	4		23.5 (7–48)	4	4
Fir HCOP Female	Dementia	3	88		3	15 (12–21)	3	3
	No dementia	6	88		6	10 (6–17)	6	2

^a^One of these patients was placed on the LCP, but taken off it 3 hours later

Characteristics of deceased patients whose case notes were reviewed

### Data analysis

Field notes from observations were written up and narratives were constructed from the case notes accessed. Research interviews were, with the permission of the participant, recorded and then transcribed. Transcriptions were checked to ensure the anonymity of participants and patients, and all data were imported into NVivo 10 for analysis. Analytic procedures were based in the constant comparative method, making comparisons at each stage of the analytic process [35]. Initial coding was undertaken and this was followed by further unpacking of the nodes. Two members of the research team worked on the analysis, and 76 % of data sources were coded separately by two individuals at the stage of initial coding.

### Ethical issues

Ethical approval was obtained from Nottingham 1 NHS Research Ethics Committee, and the authority to access the case notes of deceased patients was obtained from the National Information Governance Board. Written informed consent was obtained from participants prior to interview. For the non-participant observations a process of opting out was used, whereby all participants were given information about the study and an opt out form to indicate their wish not to be involved. Posters and flyers were also displayed prominently on participating wards.

Wards referred to in this paper have been given pseudonyms to maintain their anonymity. Patients are referred to by their study ID, family carers are referred to by their relationship with the patient and health professionals by their role and ward.

## Results

Thirty of the 42 patients whose notes were reviewed in the course of the research were placed on the LCP. All but one of the patients had in place a Do Not Attempt Cardiopulmonary Resuscitation order at the time of death, intended to prevent unnecessary and futile intervention in the event of a cardiac arrest [36]. All such orders were signed by the clinician responsible for the patient’s care, and in 14 cases it was noted that the issue had been discussed with the patient’s family. Although staff members who were interviewed were aware of advance care planning and, in some cases, saw it as something desirable for frail, older patients with multiple co-morbidities, none of them had worked with patients who had any kind of advance plan in place.

Communication between staff and carers was predominantly verbal, and mainly took the form of face to face encounters. Patients on the study wards who were approaching the end of their lives were generally beyond the point at which they could communicate with staff about their care, so the research focus moved to family carers to seek their perspectives. Three of the 33 patients whose care was observed on the wards did not have relatives who could be invited to take part in an interview. Carer and staff respondents talked about communicating with each other during the course of interviews. Carers reported variable experiences with regard to their communications with staff. There were instances of very good communication, when carers felt informed and consulted throughout their relative’s hospital stay. There were also, however, instances of poor communication when carers struggled to gain access to the information that they needed. The variability between carers’ experiences, often on the same wards, was striking.

The findings are presented here from staff perspectives first, followed by the perspectives of carers, then a case study is offered which highlights a number of the key points.

### The setting

The wards which comprised the research setting were busy, semi-public places which, particularly during the mornings, were noisy with the comings and goings of a wide range of individuals from doctors through to ancillary workers. Noise levels were exacerbated by ringing phones, patient call buzzers, voices in conversation or calling out and the sounds of equipment being moved around. Each ward had its own routines with certain times at which specific tasks were carried out such as catering staff bringing round the tea trolley, or nurses washing and dressing patients. Official visiting hours were from 2pm to 8pm but when a patient was assessed as dying their family members were allowed open visiting, so they could be on the ward whenever they wished.

The hospital has a dedicated palliative care team, including a consultant and a team of Macmillan nurses. The nurses routinely visited Ash, the acute admissions unit, to enquire whether there were any referrals for them and offer advice to staff. The other wards participating in the study had the option of referring their patients to the palliative care team, or of seeking advice from the team, but in practice this rarely occurred.

### Staff perspectives

#### Giving bad news

Acute hospital wards are oriented towards the active treatment and discharge of patients, and although death happens on a regular basis, this is not the core work of the ward. Staff, however, were confident that they could provide good end of life care, and regarded this as part of their normal work load. Informing carers that their relative is dying and has been placed on the LCP was considered the responsibility of the senior clinician, as it demands a high level of professional knowledge expertise and authority:

Although there’s always people on the ward, I don’t think they’ve always got the expertise to start talking in great detail about recognising that someone’s nearing the end of their life (Consultant, Fir).

The fact that a discussion has taken place with a patient’s family about the need to place them on the LCP was recorded in the patient’s notes, but detail was not usually provided of the content of the discussion. Each of the wards had daily multi-disciplinary team meetings at which patients and plans for their care were discussed. However, not all members of staff could be present at these meetings, and nurses described how they needed to read the medical notes of the patients for whom they were responsible on any given shift, so that they could be sure of what care and treatment was to be provided.

Consultants and other senior doctors spent limited time on the wards so they were unlikely to be available for a follow-up conversation, should the carers wish for one. One senior clinician described his practice in the following way:

My usual way of saying is, ‘Where do you think we are, and what’s been happening, what’s your understanding been?’…you have to go very gently and then you have to think about having a further discussion the next day. (Consultant, Oak)

This approach to communication with carers was unusual, both in terms of what staff participants described as normal practice and what was observed by researchers on the wards.

Once a patient had been acknowledged as dying and been placed on the LCP the major responsibility for their care passed to the nursing staff. Nurses were not so ready or able to discuss aspects of care with family carers:

I am just sort of trying to deal with any worries that the family have which usually from a nursing point of view is they will call you in if the breathing changes or they will call you in just if the patient moves sometimes even because they are, might say if the patient is in pain or whatever. But as for talking at length about conditions and prognosis, no we don’t usually do that. I think we probably will sidestep which is as well. So, no we’ll say, 'You’ll have to just ask the doctor to have a word with them', partly to do with not wanting to say the wrong thing, and partly because we do not know what to say. (Staff Nurse, Ash)

### Role of the nurse

Nurses described a different role for themselves to that of medical staff, one in which the doctor tells the family the news and then, ‘it’s the nurse’s job to support the family’ (Deputy Ward Manager, Fir). One nurse described a need to put what the doctor has said into language that the family can comprehend, demonstrating an awareness of the difficulties in understanding that carers sometimes experienced:

There’s a lot of people that will just sit and nod at a doctor and when they’ve gone, will ask the nurse. .. We had a relative last week that literally said, the doctor’s just been and told them what’s going off, I didn’t understand a word he said, can you tell me … what’s happening? (Staff Nurse, Elm)

Despite positive accounts of nursing roles and relationships with carers during interviews, observational data suggest that carers were sometimes viewed in a negative way:

Ward Manager concedes that she finds relatives a useful source of information about the patient, but does not make any reference to relatives' need or entitlement to information from staff. In fact, represents relatives as being frequently demanding and unreasonable in their manner and expectations - constantly asking for information and updates - when the patient has not changed from the previous night, or earlier in the day. Dislikes visiting times - finds relatives’ attitudes have changed: they have become more demanding, inconsiderate and rude: they are uncooperative when told not to bring flowers, to sit on beds, to limit numbers by the bedside, not to bring young children to visit (Research observation notes, Fir).

One area in which nurses’ intervention was critical concerned the initiative they took in summoning the family when they recognised that a patient was about to die:

When the curtains open PE605 is lying down with his bed flat. His head is to one side, and he is still and quiet. Deputy Ward Manager 1 comes out of the room and straight across to me. She tells me that she has phoned PE605’s wife and daughter to come in, yesterday he could talk and was with it, so there has been a rapid deterioration in his condition (Research observation notes, Elm).

This was an area in which nurses played a key role, and were frequently instrumental in allowing family members to be with their relatives as they were dying.

#### Perceived limitations to carers’ understanding

Carers were sometimes thought unable to understand and take in more than a fraction of the information that they were given:

We know you come to a medical consultation and you remember twenty percent of it, you may remember the doctor’s face, you may remember a sentence of what the doctor said…you may remember nothing at all. You go and dutifully write it all down in the notes and the relatives have a completely different recollection of what was said. (Consultant, Ash)

There was also a belief expressed by some staff that when families did not anticipate that their relative was approaching the end of life, one conversation with the doctor was insufficient to allow them to understand that their relative was dying and what the implications of this might be. One staff nurse expressed this by saying:

I think there’s probably levels of acceptance, and maybe as nurses and practitioners and doctors, we think, because we’ve had one conversation where we’ve said very clearly, He’s coming to the end of his life, and it could just be a matter of days now…and they nod and they say Yes, okay, that doesn’t mean to say that that’s been fully accepted or processed by them…you might need to have the conversation again, but obviously, nurses and doctors don’t want to have that conversation every day, and you think if it’s happened once, why would you go there with someone? (Staff Nurse, Oak)

Such a perspective puts the onus of understanding on the carers, implying that health professionals have carried out their duty by having the one conversation with the family and that if the family wish for more it is their own responsibility to seek that out. However, members of staff understood cues that a patient was thought to be dying which to carers were obscure and incomprehensible. Cues such as the cessation of routine observations or the permission given to carers to visit at any time, were obvious to health professionals as markers that a patient was dying. Some respondents also felt it should be obvious to families that their relative was dying:

It’s pretty obvious that somebody’s dying because, you know, the sombre lighting and the sombre staff and other people coming in and focusing on symptoms rather than, you know, strutting around with charts and drug cards and you know. (Consultant, Ash)

It seemed difficult for staff to remember that the terrain of the ward and its habits and processes which were so familiar to them were alien to family carers, who were often unable to understand what was happening to their relative. It was rare for a member of staff to explain to carers in any detail what they might expect as their relative was dying, perhaps through a failure to appreciate that carers had little or no experience with dying. Lack of contact between family carers and members of staff prohibited the establishment of mutual understanding. It was not unusual to see, while carrying out research observations, that staff did not greet relatives when they arrived on the ward and often moved about the ward with downcast eyes to avoid eye contact. Indeed, the missing or avoidance of opportunities by staff to communicate proactively was a striking feature of observations. Staff seemed to exhibit a reluctance to engage with the relatives of patients, while family members appeared uncomfortable and ill at ease when visiting their relative; such discomfort could contribute to an unwillingness to approach staff for information.

### Carers’ perspectives

Carers who were interviewed had varied experiences. When the dying patient was placed in a side room it was easier for their family to spend time with them without being disturbed by the routine work of the ward. However, when patients remained in the open bay visiting could be problematic, both for the family and for the wider population of the ward. Spending time with a dying patient while the routine work of the ward went on was potentially distressing for family members, some of whom were also concerned about being a source of discomfort for other people on the ward who might realise that their relative was dying and consequently be distressed.

The family carers of two patients reported having very good relationships with staff on the ward. In both cases they had talked several times with the consultant who was caring for their relative, they felt accepted by ward staff and they encountered no difficulties in accessing the information they needed. Three family carers reported very poor relationships with staff, to the extent that they felt abandoned and unsupported. In two cases carers struggled to find a member of ward staff to inform when their relative died. Most carers, however, reported an experience that was somewhat neutral; their expectations were low so they were not disappointed, or they made excuses for what might otherwise be considered shortcomings in staff communication with them.

#### Receiving bad news: Clarity

Recognising that their relative was dying was a critical point of transition for carers. Some respondents expected their relative’s death. In other cases, when it came, the death was sudden and unexpected. For others it was a gradual process of realisation as their relative became increasingly ill and frail.

Carers came to the understanding that their relative was dying in a number of different ways. In conversation with the doctor one daughter was told bluntly that her mother was dying imminently:

Right, she says, ‘What, what I’ve got to tell you isn’t nice’, she says, but, she says, ‘Your mum’s stomach has rotted…’, I says, ‘Right.’ So she says, ‘So, she needs an operation. To save her life.’ So I says, ‘Okay.’ So she says,’ But, because we can’t stabilise her diabetes.’ she says, ‘We cannot operate, she’s too weak.’ So I says, ‘Okay.’ She says, so I’m thinking, ‘Okay, so, they can’t operate now, they’ve got to get, sort the diabetes out.’ So, I says, ‘So, what happens from here?’ So she says, ‘Well, if we don’t operate, your mum will die.’ So I says, ‘Fair enough.’ I says, ‘So, how long do you reckon my mum’s got then, without the operation?’ She says, ‘An hour, maybe two.’ Well, I was devastated. (Daughter of PNCA402, an 83 year old female patient)

The bluntness of this communication made a strong impression on the patient’s daughter. It left her in no doubt that her mother was dying, but added to the distress involved in this experience.

Some carers came to an understanding that their relative was dying when their suspicions were confirmed by professionals. One daughter, for example, spoke about how she had ‘thought he (her father) looked like somebody who was dying’, which made her receptive when the doctor told her that this was the case:

and the doctor had pre-warned me that the end of somebody’s life through fibrosis isn’t pleasant both for any party…on the night that he was dying, I thought they were very, very good…when we came, obviously, they knew when we came in, what the situation was. (Daughter of PNCO206, an 86 year old male patient)

This respondent felt included in decisions about her father’s care and thought that ward staff were supportive and understanding, as did this daughter:

And dad hadn’t been eating and drinking for a long while and there was problems again with his aspiration so he, he (consultant) felt really that he was at the end of his life so we, we took a decision to withdraw… any sort of feeding… you know, it was dad’s time, and really, it was dad’s time. (Daughter of PNCO203, an 85 year old male patient)

This patient died during his second admission to Oak ward. His wife and daughter had visited regularly and had helped him with his meals. They both felt that they got to know ward staff and that they were fully consulted about all decisions regarding his care:

Because he spent nine weeks on the ward, from the end of April, we knew them (staff) so well and I was going at lunchtime to help with feeding him and…we just knew the staff so well and they were so caring. (Daughter of PNCO203, an 85 year old male patient)

#### Receiving bad news: Lacking clarity

Not all family carers knew that their relative was assessed as dying, and thus the death was sudden and unexpected. Some respondents reported that they had either not been told explicitly that their relative was dying, or they had not been told in a way that enabled them to understand or accept this news. Sometimes it was only through a process of retrospective reflection that carers realised the significance of events and interactions with staff. One female patient’s partner, for example, initially thought that he had not been told how ill she was, but he decided during the course of the interview that perhaps he had been told but had forgotten, or not realised the significance of what had been said at the time:

I’m not too sure when I actually realised it, when it hit me when I was on my way back to the house, but I’m not sure quite when I realised it, yeah. I think they did actually. I think they did. Because I had a little chat with them at one point. And, (…) I think they did tell me that actually … (Male partner of PNCA401, a 96 year old female patient).

This participant was not alone among carers in being unsure about what the family had been told, or in failing to interpret this in the way intended by staff. The wife of one patient was told that he was dying and understood what she was told. However, she and her husband had been through similar experiences before and this prior experience led her to believe that he would survive on this occasion, too:

(she said, he’s) ‘really poorly’. I said, ‘Yes, clearly, I realise that, we’ve been through this many times before.’ And she said ‘Well, we don’t think he’ll pull through this time.’ So, that was a shock… So, anyway, about three o'clock in the morning, he kept waking up, and was sitting holding his hand and he was squeezing my hand really hard and I thought, No, this isn’t what a dying man is, he’s too strong, he’s fighting this again. (Wife of PNCA403, a 63 year old male patient)

Her confidence in her husband’s survival was so strong that she went home, and so was not with him when he died which was devastating for her. Prior experience of occasions when her husband had almost died prevented her from fully assimilating the information she was given about her husband’s prognosis. In a highly charged emotional state, it is understandable that carers may fail to comprehend what is said by staff, particularly if this is framed in vague and euphemistic terms. There is a tendency to construe developments in line with desired outcomes, and to place greater reliance on the concrete evidence of past experience rather than the anticipation of professional judgement. In addition, the way in which staff offer information in a vague and sometimes equivocal fashion exacerbates this situation.

Carers could misunderstand what ward staff said, as sometimes staff spoke to family carers in ways which were ambiguous and with meanings that were unclear to the carers. One patient’s wife, for example, was told that she could visit her husband outside scheduled visiting hours, but the implications of this were not explained to her:

He…was always asleep when I went. And the nurse said, ‘Well, you can come in, [he’s] bit more alert in the morning, come then.’ I ought to have just [realised], alarm bells rang, but anyway, he woke up then, he looked at me, says, ‘Hello duck’, smiled, went back to sleep. (Wife of PNCO201, an 85 year old male patient)

Later his wife realised that she had been given more access to her husband because he was dying, and she blamed herself for not understanding the situation.

What seems to be critical in family carers’ understanding that their relative is dying is the alignment between staff and carer perspectives. PNCO201’s wife realised that he was approaching the end of his life, but believed that he had another one or two years of life. PNCA401’s partner and PNCA403’s wife quoted above were not receptive to the prospect that death was imminent, and did not easily accept or internalise the messages conveyed by staff. In contrast, the daughters of PNCO203 and PNCO206 had already realised that their fathers’ deaths were likely to occur soon, and consequently were receptive to what they heard in their discussions with staff on the ward. When there was a lack of clarity in communication, whether through the use of euphemistic or vague language, this could lead carers to develop or perpetuate expectations which health professionals would realise were unrealistic in the event that carers’ understandings were successfully explored.

#### Seeking a hearing

It was not always easy for carers to find someone on the ward to whom they could speak. Doctors, particularly the consultants who have the requisite authority to divulge a poor prognosis, were not present on the ward all the time and their schedules often do not fit with visiting hours:

Staff Nurse1 was going round the bay with the observation machine, she gets into quite a tetchy argument with B4’s^1^ male visitors. They are trying to find out what is going on with their mother. Apparently they had had a phone call from a doctor yesterday and they are keen to have another talk with him. They are trying to get Staff Nurse1 to arrange an appointment with the doc for them – Staff Nurse1 is irritated about this and tells them that if they want to see the doctor they will have to come on the ward and gives them some very general times when they might coincide with the doctor’s presence and availability. Male visitors are not happy with this – clearly feeling that the doc should be more accessible… The male visitors are quite persistent. They can’t believe that it is necessary for them to make a special trip to the ward in order to see/talk to the doctor… The situation does not seem to be resolved before Staff Nurse1 manages to terminate the encounter and move on (Research observation notes, Fir).

In addition, family carers were not always sure who it was they needed to speak to about a particular issue:

I wouldn’t have known who to ask. And there wasn’t really, I mean, the doctors were so busy, so, you know, trying to see a doctor and stuff, it was a bit difficult. (Daughter of PNCO202, a 75 year old male patient)

Although nurses’ work roles required them to be present on the ward most of the time, carers could still find it difficult to find a nurse who was able to answer their particular query. The effort to find a member of staff to talk to could also be a source of frustration for the carer:

Only because, you could never get hold of anybody. I mean, they were so busy. I mean, sometimes you’d think, 'Where are they?' (Daughter of PNCO206, an 86 year old male patient)

Staff tended to have little contact with carers during their visits to the wards, with the onus placed on carers to seek out a member of staff if they wished for information. Doctors, like nurses, could be difficult to pin down:

Doctors huddle in 2s and 3s to talk in the corridor – this enables visitors to target them for a talk and information - though sometimes have to lurk for a while until they succeed in getting their attention. My impression is that they tend to be fobbed off, and that the doctors don’t seem to be forthcoming with information… On his way up the ward a pair of relatives from another bay collar him for information about their relative’s discharge tomorrow – he is rather brusque and unhelpful, as far as I can see. Later on the couple manage to engage a nurse who is evidently much more constructive and useful in her response… The older woman tries to engage with a doctor – but is rather rebuffed, I thought, as he turns quickly to leave the ward (Research observation notes, Ash).

Carers tended to perceive staff as being very busy, and lacked a sense of entitlement or confidence in approaching staff members directly or pursuing their wish for information. Family carers tended to wait for information to be offered to them, but reported this did not often happen.

Carers’ assessments of staff communication were also shaped by their expectations of staff behaviour, and carers were keen not to appear too critical:

I think my husband went back out and said to the lady on the desk, ‘Can I speak to the nurse who’s looking after her?’ And she said, ‘The nurse has gone on her break.’ So I said to my husband at the time, ‘Well, everyone has to have a break, you know?’ [laughs] That is the way I look at it, everyone has to have a break. It, we weren’t complaining because she wasn’t there, it just would have been nice if, if the lady who’d gone on her break had said, ‘Can you greet Mr and Mrs S and, you know, reassure them.’ (Niece of PNCF801, a 91 year old female patient)

Several respondents described the demeanour of ward staff as being distant and clinical, but qualified any implied criticism by explaining that this was what they expected and consequently was not considered to be inappropriate or lacking.

#### Misdirection from external cues

In some cases carers had mistaken views about what was happening with their relative because they misinterpreted cues that they observed in the immediate environment. One daughter was pleased when she learned that her mother had been moved from a side room onto the open ward. She said:

When I left her, she was in a wa- in a room on her own. When I got back, I was just going back to the room, and they said, ‘Oh, your mum’s been moved down into the ward.’ I thought, Oh, this is good news. (Daughter of PNCA402, an 83 year old female patient)

However, the reality was that, even though she was recognised to be dying, the pressure on beds resulted in the patient being moved out of the side room to make way for another patient who required isolation because of infection.

#### Feeling abandoned

Some family carers, when their relative came close to death, described feeling abandoned by staff on the ward. PNCA402’s daughter, quoted above, described how she and her family were left alone with her dying mother on the busy ward during regular visiting hours. She was aware of other families visiting their relatives and asked a nurse if she could close the curtains round her mother’s bed. She went on to say:

Nobody came to see her after that. Nobody came to see if we was okay. Nobody, nobody even come to explain to, such as my brother and the rest of the family, why is this happening. It was just left to me. And, and then, my mum died within the hour. (Daughter of PNCA402, an 83 year old female patient)

When she recognised that her mother had died, the patient’s daughter rang the bell but no one came. Eventually she went to find a nurse and inform her that her mother had died. The partner of a male patient had a similar experience, when she and her partner were left alone as he was dying. She, too, struggled to find someone to inform once he had died:

But he didn’t take more than like, less than a minute, less than half a second, so I pulled the curtain round and I sit with him for five minutes til he completely take the last breath. But the problem is, what irritates me, because I couldn’t find anybody. Because the time …at that time, I just need somebody to be there. (Partner of PNCO205, a 68 year old male patient)

The cases described here illustrate a lack of awareness on the part of staff of the emotional distress that carers keeping vigil for dying patients on the wards may experience. However, carer discontent is defused by the low expectations that they have of how staff should behave. They often make allowances, expressing their awareness that staff are very busy, and describing their own negative responses as inappropriate. Carers tend to have a low sense of entitlement or expectations of support. This also illustrates the collaborative work that relatives do to help the ward run smoothly, by containing and controlling their emotional reactions and minimising the demands they make on staff.

### Case study

The following case study highlights a number of the communicative issues as experienced by carers, consideration of which will be picked up in the Discussion section below.

PO104 was admitted with severe dementia and an infected wound. She developed kidney failure and pneumonia. A DNACPR order was signed and three days later it was recorded in her notes that care was primarily palliative. One of the doctors spoke to her family, informing them that her prognosis was ‘very guarded’ and he recorded in her notes that they understood. A few days later the doctor spoke to the family about LCP; they were against stopping treatment and at this time the doctor decided to continue active care. Open visiting hours were in place for PO104’s family. Consultant recorded ‘I suspect ≤50/50 (survival) regardless of what we do’. PO104 had a buprenorphine patch to help alleviate pain, but her family were recorded in her notes as disapproving of this. The ward recorded that after discussion the patient’s son ‘seemed to accept this’, but family stated during interview that they did not agree to its use. The possibility of the patient’s being fast tracked home to die was discussed and explored, but did not happen. Three weeks after admission PO104 was placed on the LCP, and according to her medical notes her daughter in law was informed. At this point she was to be treated symptomatically and all observations were to be stopped. The following day it was recorded in PO104’s medical notes that the doctor had talked with the family about LCP, and that they wanted her to be given fluids but not analgesia. PO104 died four days later, and her family arrived on the ward shortly after her death. During an interview with PO104’s son and daughter in law it was clear that they did not understand that she had been placed on the pathway with the goal of ensuring she was kept comfortable throughout the inevitable process of dying, and they thought that she was being actively treated until the moment of her death. While PO104 was on the LCP, her daughter in law queried with a doctor why nurses were no longer carrying out observations. The doctor told her that it was no longer necessary to do so, but the carer thought the doctor was simply ‘sticking up for’ the nurses, and their poor care. There was a high level of misunderstanding between what the family were told, what they understood and what medical and nursing staff thought the family understood. PO104’s family were unaware of the DNACPR order and would not have agreed to it. Staff members who were interviewed and spoke about this family described them as not being ‘on the same page’ as staff; as being ‘in denial’; as being ‘a little bit strange’.

## Discussion

The wards which participated in this study were busy, noisy spaces with much activity and many people, all of which made communicating about confidential matters difficult. There was variation in the level and quality of communication, with some good and some poor examples observed. There were different consultants working on the four wards, who had different levels of skill and experience in approaching the topic of end of life care, with both patients and family carers. On the whole staff were aware of whether or not they were communicating well with patients and carers. There were a number of factors which may have prevented them from being more effective, such as a fear of saying the wrong thing, lack of confidence in communicating the uncertainty which often accompanied a patient’s prognosis and the institutional nature of an NHS hospital. The culture of the NHS on a national level encourages efficiency. Measurements and indicators of efficiency do not include the amount of time staff members spend talking to patients and their families.

Carers who participated in the study had differing experiences. Some, such as PNCO205’s partner and PNCA402’s daughter, had a poor relationship with staff from the time when their relative was admitted and by the time of their relatives’ deaths they felt completely abandoned. Others, such as PNCO203’s wife and daughter, had a positive experience and felt as though they were fully informed and involved in decisions about care. They had a good relationship with staff which had built up during a previous, extended, hospital stay. Most carers, however, had a mixed experience while their relative was on the ward, but their low expectations led them to make allowances for staff whom they perceived as too busy to spend time talking to carers. Carers in this situation were told the bad news about their relative, but this tended to be done in vague terms or using euphemistic language which was open to various interpretations. Carers would then interpret what they were told through the lens of their previous experience and knowledge, which could leave them expecting a better outcome than was perhaps intended, as was the case for PNCA403’s wife.

Carers of dying patients often struggled to make sense of what was going on as once the initial telling of the bad news had taken place they encountered difficulties in locating a member of staff from whom they could seek clarification or further information, or with whom they could discuss their worries. At this stage staff did not make themselves easily available to carers. Carers were seen to struggle to retain the information they were given or to fully comprehend the meaning, particularly when euphemism, jargon or vague language was used. This was particularly the case when staff were attempting to break potentially painful news gently by using euphemism or roundabout language. However, instead of talking to carers on more than one occasion, to reinforce a message, or elicit concerns and possible misunderstanding, staff tended to avoid engaging with them. The staff nurse on Oak ward quoted above illustrates how some staff recognised the desirability of having more than one conversation with carers, but also acknowledged the reluctance on the part of staff to talk about dying if it could be avoided.

Sometimes, also, carers were criticised for having unrealistic expectations with regard to their relative’s survival. For example the family of PO104, the patient described in the case study, were criticised by staff as being ‘strange’ and ‘in denial’ for the way in which they interpreted the information they were given and for the opinions they expressed about possible outcomes for PO104. Events made it clear that PO104’s family did not realise how close to death she was, as when her daughter in law asked why the nurses had stopped carrying out observations. Members of staff were thus presented with opportunities to engage in further discussions with members of the family and clarify their understanding of PO104’s situation, but these opportunities were not taken.

Medical and nursing staff have a professional duty to communicate effectively with carers [19, 20]. Some nurses said during interview that they took on a role in which they provided ongoing support for families, and that they would interpret what the doctors had said in such a way that the carers could understand it. This was in contrast to the reports that carers gave during interview and to observations made by researchers on the wards, but it is not unusual for research participants to give idealised accounts of behaviours that are not consistent with practice [37]. The observation that lay people do not always remember or understand what they are told by health professionals belongs to a mechanistic model of communication. In such a model the purpose of communication in the healthcare setting is to educate the listener, and this occurs through the passing of information from professionals to carers. Carers in this model are viewed as more or less passive recipients of the professionally-directed message [38].

Data from this study supports the view that this is not an effective way of communicating with carers. Information is not effectively passed on and carers do not have the opportunity to discuss their expectations and worries. Carers were often given vague or equivocal information which they were then left to interpret and, as was the case with PNCA403’s and PNCO201’s wives, they may do this in ways unanticipated by staff. Such a mechanistic perspective to communication makes no allowance for discussion nor for engaging with the prior understandings and experiences that a lay person has. If the message fails to achieve what was intended by its instigator this failure is not attributed to poor communication skills on the part of the health professional but to carers’ lack of understanding and their inability to retain the information that they are given [38].

An alternative model of communication is needed which allows professionals to pass on their understandings, but also allows carers to engage with what they are told and contribute their own perspectives. Standard ‘breaking bad news’ guidelines include checking for understanding. The observations suggest this is often not done. In addition it appears that both staff and relatives lack a common framework for communication (e.g. staff not checking prior knowledge, not seeking what information relatives want, fearing relatives’ responses, ‘getting it wrong’ or criticism; and relatives having to seek and initiate contacts, lacking the confidence or skills to manage the encounter in a dis-empowering setting). In addition staff uncertainty and lack of time make communication difficult. One model of communication which endeavours to overcome the power differential that exists between clinicians and patients or carers is concordance [39]. This model developed out of an inquiry carried out for the Royal Pharmaceutical Society of Great Britain during the 1990s, motivated by the desire to understand why patients do not always take medication in the way that is intended by the physician. Concordance involves an attempt to move away from the traditional paternalism that was inherent in the notion of compliance in relation to medicine taking, in which the patient was simply expected to follow the physician’s advice [40]. A concordant model of communication views the consultation between clinician and patient as one in which a negotiated exchange takes place, in which the patient is able to participate fully and share her or his perspectives on offered diagnosis and treatments [39]. The development of concordance theory in relation to professional and lay healthcare relationships drew on existing theories such as Tuckett et al.’s theory of the medical consultation as a meeting between experts [41], and Kleinman’s work on explanatory models in effective communication between patients and healers [42]. Although early work focused upon interaction between physician and patient regarding the use of medication, concordance has a wider application as a model of shared communication and decision making in medical consultations more generally. The key point to a concordant consultation is that each party reaches awareness, understanding and respect for the other’s perspective, and uses this as the basis for negotiation and agreement. Transferred to the conversation between health professionals and family carers, a concordant consultation could enable a negotiated understanding to be reached about the current status and future prognosis of frail, older patients approaching the end of life.

Such a concordant approach to communication between staff and carers would permit negotiated understandings to be reached. Each party to the discussion would be enabled to understand the other’s perspective and mutual respect and awareness would lay the foundation for agreeing a course of action. Such a process does not deny the clinician’s expertise nor detract from his or her duty to provide the best care for the patient, but it does allow for insights that the carer has to be incorporated into the plan for care [39, 40]. A concordant approach to communication also enhances the likelihood that carers will both understand and agree with any decisions made with regard to their relative’s end of life care [28, 29]. However, this approach needs to take account of the considerable uncertainty that often exists about the approach of the end of life (which can be acknowledged and discussed), and the fast-moving nature of acute healthcare, with sometimes rapid deterioration (or recovery), short lengths of stay, and lack of a prior relationships between staff and families.

Findings presented in this paper demonstrate marked continuity with the findings of much earlier research, suggesting that communication is a difficult skill to master. Healthcare professionals have been reported as unwilling to talk openly with patients and their families about death and what is likely to happen as someone dies [43, 44]. Nurses have been reported as uncomfortable about caring for those who are dying [32]. Carers of hospitalised older people have been found to struggle to obtain information from professionals. Some members of staff caring for older patients on acute hospital wards, the same kind of setting as in this study, have displayed a tendency to mistrust carers and avoid engaging with them [7]. Despite an emphasis on the importance of good communication between health professionals and patients and their families [19, 20, 25] there is still a similarity between the lack of open awareness which Glaser and Strauss identified in the 1960s and the lack of awareness described by some families in this study [44].

## Conclusions

This paper has described the communicative processes found in operation between healthcare professionals and the family carers of dying patients on four acute care wards in a general hospital. Hospital systems and processes were not designed with the expectation that communication would play a central part in the experience of care, with communication being given low priority, and the environment being unconducive to sensitive discussions. Members of staff rarely made much effort to engage with family carers, to elicit their understandings about their relative’s health status, or to address their concerns. However, some staff members did take the time to engage with carers in this way, and some families therefore had a much better experience of care than others. Although it is not only professionals who contribute to the communicative process they have the responsibility for ensuring effective communication with the family carers of their patients. Carers make a significant contribution to communication and it is this joint effort which determines the outcome. The nature of the understandings and expectations that carers bring, and the skills that they possess, can impact strongly on the outcome of a communicative encounter. More research is required to explore the dynamics of the interactions that carers have with staff in the acute hospital setting, and to identify those factors which contribute to family carers having a better experience of care while their relative is dying on an acute hospital ward.

## Abbreviations

DNACPR: Do Not Attempt Cardiopulmonary Resuscitation
LCP: Liverpool Care Pathway for the Dying Patient
NHS: National Health Service
UK: United Kingdom

## Footnotes

1 Bays on the wards were given colours as names, and each bed with the bay was given a number. Individual bed spaces were identified by the bay colour and bed number, in this case Blue bay, bed 4, hence B4. Referring to patients by bed number and bay here, while impersonal, preserves the patient’s anonymity.
